# Failure of lipid control by PCSK9 inhibitors in compound heterozygous familial hypercholesterolemia complicated with premature myocardial infarction: A case report

**DOI:** 10.1002/ccr3.8498

**Published:** 2024-03-14

**Authors:** Ziyue Zhang, Rongpei Yang, Jun Zhu, XiaoLi Yang, Hao Luo, Hongyong Wang, Xiaoli Luo

**Affiliations:** ^1^ Department of Cardiology, Daping Hospital The Third Military Medical University (Army Medical University) Chongqing P. R. China; ^2^ 96608 Hospital of PLA Han Zhong Shanxi P. R. China

**Keywords:** familial hypercholesterolemia, low‐density lipoprotein receptor, myocardial infarction, PCSK9 inhibitors

## Abstract

**Key Clinical Message:**

A certain level of low‐density lipoprotein receptor activity is crucial for the efficacy of PCSK9i. Therapeutic strategies for familial hypercholesterolemia patients should consider drug efficacy, and genetic testing will be helpful.

**Abstract:**

Familial hypercholesterolemia (FH) is a serious autosomal dominant disorder. Managing blood lipids in FH patients poses greater challenges for clinicians. Drug therapy may not always yield satisfactory results, particularly in individuals with low‐density lipoprotein receptor (LDLR) negative mutations. Herein, we report a young female harboring an LDLR frameshift mutation. This patient developed xanthomas at 7 months old and underwent several years of treatment involving four classes of lipid‐lowering drugs, including PCSK9i. However, the response to drug therapy was limited in this patient and eventually culminated in premature myocardial infarction. The efficacy of PCSK9i depends on the activity of LDLR. The inefficacy of PCSK9i may arise from the extensive mutations which leading to loss of LDLR activity. Therapy plans for these patients should take into account the efficacy of drug therapy. Early genetic testing is crucial for clinicians to make informed decisions regarding therapy options.

## Graphical abstract

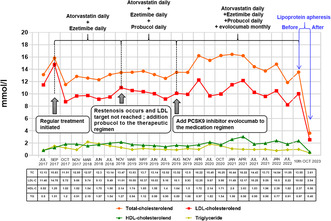

## 1 INTRODUCTION

Familial hypercholesterolemia (FH, OMIM # 143890) is an autosomal dominant disease characterized by markedly elevated levels of low‐density lipoprotein cholesterol (LDL‐C). The consequences of FH are severe, including the presence of multiple tendon xanthomas throughout the body, and a heightened risk for coronary heart disease and myocardial infarction.^1^ The majority of FH cases arise from mutations in genes encoding low‐density lipoprotein receptor (LDLR), apolipoprotein B (ApoB), or proprotein‐converting enzymes/kexin9 (PCSK9).^2^ Effective management of LDL‐C levels plays a crucial role in determining the prognosis of FH patients.^3^ PCSK9 inhibitors (PCSK9i) are recommended as lipid‐lowering therapies in patients with FH to further reduce cholesterol levels in conjunction with drugs such as statins or ezetimibe.^4^ However, not all FH patients respond well to PCSK9i. Here we present a case of compound heterozygous FH, where standardized therapy including PCSK9i failed to control LDL‐C levels, ultimately resulting in premature myocardial infarction.

## 2 CASE PRESENTATION

In 2017, a 21‐year‐old female patient presented to the cardiology department with complaints of recurrent chest tightness and crushing retrosternal chest pain. These symptoms typically occurred during physical activity and subsided after rest. Each episode lasted approximately 3–5 min. The aforementioned symptoms recurred frequently and were primarily triggered by exertion. The patient denied experiencing dizziness, nausea or vomiting, shortness of breath, diaphoresis, or any other associated symptoms. The onset of these symptoms dates back to 10 years ago and has progressively worsened over the past week.

Xanthelasma measuring approximately 2–3 cm in size emerged adjacent to the gluteal sulcus, when the patient was 7 months old (1997). Subsequently, there was a progressive increase in both quantity and size of yellow plaques and nodules. From ages 3 to 12 (1999 to 2008), this patient underwent multiple treatments for yellow skin patches at the dermatology department, including xanthoma resection and biopsy. The postoperative histopathological examination of the nodules in both the left and right foot confirmed the diagnosis of xanthomatosis. The blood lipid levels of this patient at the age of 7 (2003) were as follows: total cholesterol (TC): 19.37 mmol/L (normal TC: <5.2 mmol/L), low density lipoprotein cholesterol (LDL‐C): 16.68 mmol (normal LDL‐C: <3.4 mmol/L). However, no lipid‐lowering treatment was administered. At the age of 12 (2008), this patient received statin therapy for lipid lowering after being referred to the endocrinology department. Nevertheless, she did not take medication regularly and lacked consistent follow‐up or therapeutic interventions.

**FIGURE 1:**
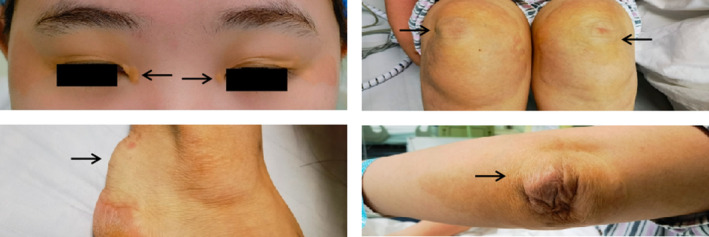
The patient exhibits xanthoma in the ocular, patellar, Achilles tendon, and cubital fossa regions (after surgery).

A physical examination findings were as follows: body temperature: 36.5°C, breathing rate: 19 beats/min, heart rate: 79 beats/min, blood pressure: 120/81 mmHg, and blood oxygen saturation level: 98%. The patient presented with raised yellow plaques on the upper and lower eyelids, both elbows, both knees, and the Achilles tendons (Figure 1). Laboratory parameters revealed elevated TC levels of 15.83 mmol/L (normal TC: <5.2 mmol/L) and LDL‐C levels of 14.78 mmol/L (normal LDL‐C: <3.4 mmol/L). Myocardial injury markers, B‐type natriuretic peptide (BNP), fasting blood glucose levels, liver and kidney function tests, routine blood tests, and coagulation factors were all fell within the normal reference range.

Next, the patient underwent coronary angiography, revealing diffuse stenosis of approximately 40%–90% throughout the entire course of the right coronary artery, TIMI 3 flow. The most severe stenosis was observed in the proximal and distal segments. The left anterior descending coronary artery exhibited diffuse narrowing of about 40%–90%, a myocardial bridge was observed in the distal segment with approximately 99% systolic stenosis, TIMI 3 flow. An aneurysmal change with localized dilation was noted in the proximal circumflex branch, which had a stenosis of around 40% (TIMI 3 flow). Consequently, percutaneous coronary intervention (PCI) was performed on July 3, 2017 for the left anterior descending artery and on July 14, 2017 for the right coronary artery.

**FIGURE 2:**
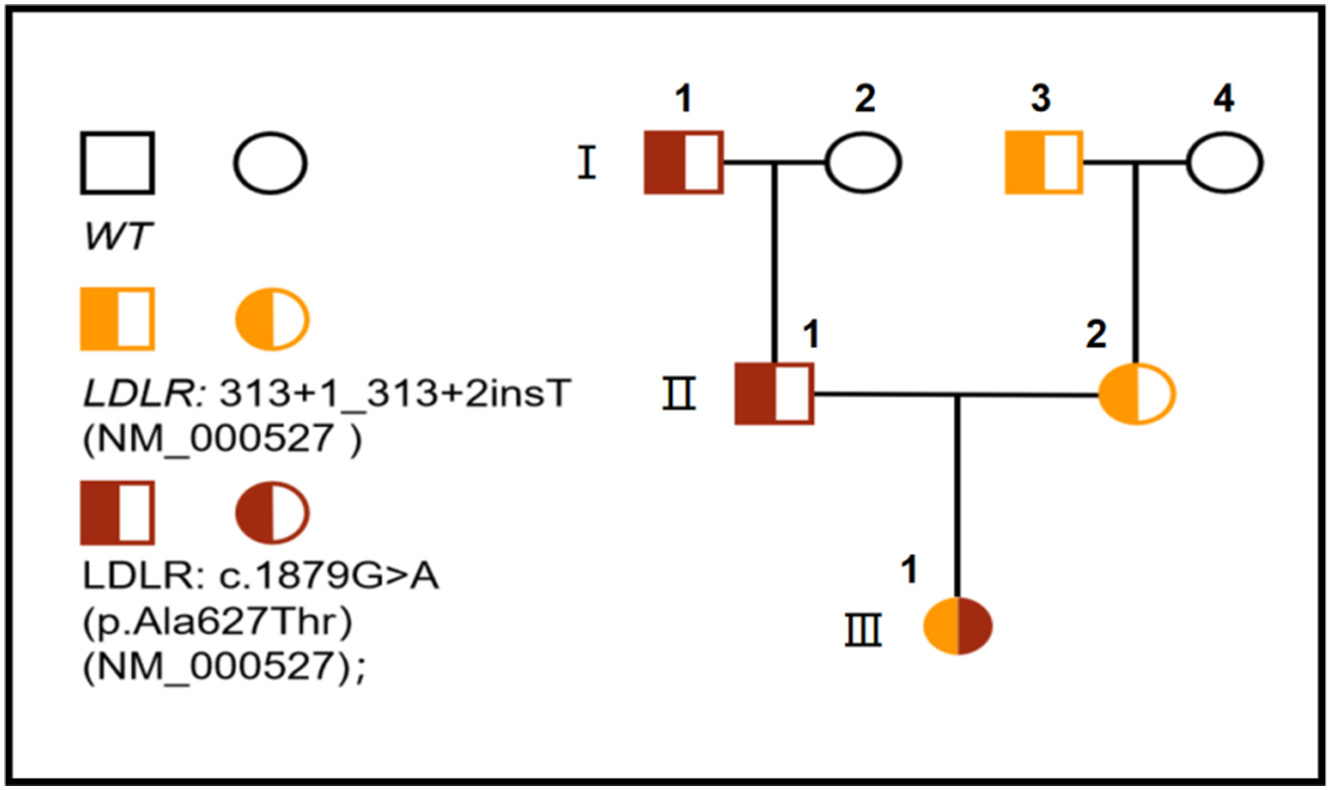
Pedigree analysis of the patient's family was conducted.

The patient's symptoms exhibited significant improvement following treatment. According to the simplified clinical classification criteria proposed by the American Heart Association guidelines, this patient received a clinical diagnosis of homozygous familial hypercholesterolemia (HoFH). To further elucidate the diagnosis, genetic testing was performed on both the patient and her immediate family members, with their consent. Sanger sequencing and spectrometry method verification revealed two mutation sites in the proband's LDLR gene. One of these mutation sites, c.313+1_313+2insT, was found in her mother's chromosome 19 LDLR gene sequence NM_000527 and has not been previously reported. The other mutation site is a c.1879G>A point mutation in the father's chromosome 19 LDLR gene NM_000527 (Figure 2). Based on these findings, this patient was diagnosed with compound heterozygous FH. Compound heterozygosity is considered to have a clinical severity comparable to true homozygosity.^5^

**FIGURE 3:**
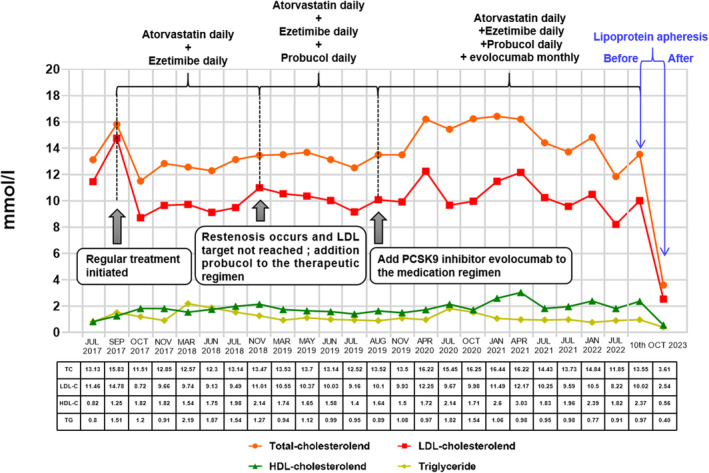
The lipid levels of this patient were monitored over a 6‐year follow‐up period, and the values are reported in mmol/L.

Subsequently, the patient received treatment with atorvastatin calcium tablets (40 mg qd) and ezetimibe (10 mg qd). Additionally, lifestyle interventions encompassing dietary recommendations and regular physical activities were implemented as part of the therapy plan. Afterward, we conducted long‐term follow‐up assessments of the patient's blood lipid levels and generated a waveform diagram based on the blood lipid levels and lipid‐lowering treatment strategy (Figure 3). At the subsequent follow‐ups, the patient's blood lipid levels were far from reaching recommended guidelines. Therefore, probucol (125 mg bid) was added to the patient's lipid‐lowering strategy. However, even after further follow‐up evaluations, the patient's blood lipid levels still did not meet the desired control goal.

In July 2019, at the age of 23, PCSK9i (evolocumab 420 mg/month) was incorporated into the patient's drug regimen. Despite this addition, it is perplexed that her blood lipid levels did not decrease and even rebounded. Ultimately, we had to resort to a specific technique, lipoprotein apheresis. However, this patient refused. Furthermore, lipid‐lowering drugs which do not rely on LDLR present a promising alternative treatment for this patient.^6^ Examples include lomitapide and mipomersen, which contribute to lipid reduction by inhibiting microsomal triglyceride transfer protein (MTP) and ApoB100 synthesis.^7^ Unfortunately, lomitapide and mipomersen are not accessible in China mainland areas, thus we ultimately abandoned these options. Not surprisingly, due to inadequate blood lipid control, the patient presented our chest pain center with sudden and severe chest pain in 2021 at the age of 25. The diagnosis confirmed non‐ST‐elevation myocardial infarction. In October 2023, this patient chose to undergo lipoprotein apheresis ultimately, resulting in a significant reduction in blood lipid levels (Figure 3).

## 3 DISCUSSION

FH patients have been shown to exhibit favorable response to PCSK9i, resulting in effective reduction of blood lipid levels. Few cases have reported inefficacy of PCSK9 inhibitors. However, the average LDL‐C value for this patient remained unchanged at 10.38 mmol/L (400.67 mg/dL) after treatment with PSCK9 inhibitors, compared to 10.34 mmol/L (399.12 mg/dL) prior to treatment initiation. The LDL‐C level in this patient significantly exceeds the target level recommended by the European Atherosclerosis Society (EAS), <1.8 mmol/L (<69.5 mg/dL).^8^

The classification of FH includes heterozygous familial hypercholesterolemia (HeFH), characterized by single allele mutations, homozygous familial hypercholesterolemia (HoFH), characterized by identical allele mutations, and compound heterozygous FH, characterized by different mutations in each allele.^9^ According to the genetic testing results, mutations of this patient are derived from different mutation sites inherited from her parents, thus she should be classified as a compound heterozygous HF. It is worth noting that the disease severity of compound heterozygosity is comparable to homozygotes.^5^ Typically, the total cholesterol levels of HeFH patients range between 8 and 15 mmol/L, and most of them experience coronary heart disease before 55–60 years old; however, HoFH patients generally present with total cholesterol levels approximately between 12 and 30 mmol/L and experience earlier onset of coronary heart disease.

Different mutations are believed to potentially impact the therapeutic efficacy of drugs in FH therapies. Generally, LDLR mutations are considered to exhibit the most severe clinical symptoms and the worst therapeutic response compared with ApoB and PCSK9 mutations.^9^ Approximately 80% of FH patients are attributed to LDLR mutations, with over 2000 mutations have been identified.^10^, ^11^ LDLR can be categorized into defective mutations and negative mutations based on the extent of mutation and preserved function. The function of LDLR in negative mutations is less than 2% of normal levels, while defective mutations retain a function ranging from 2%–70% of normal levels.^12^ The degree of preserved LDLR function correlates with the severity of FH and response to drug therapy. Patients harboring LDLR negative mutations generally present higher levels of LDL‐C and have a worse prognosis compared to those with defective mutations.^13^

The efficacy of lipid‐lowering therapy in patients with HoFH and compound heterozygous FH is heavily dependent on preserved LDLR activity.^14^ This may account for the lack of response to PCSK9i in some patients. LDLR reduces plasma LDL‐C levels by binding and internalizing LDL‐C, while PCSK9 prevents LDLR from recycling to the cell surface by binding to LDLR and forming a complex that enters lysosomes for degradation. Inhibiting PCSK9 with PCSK9i can preserve LDLR on the cell surface and increase the uptake of circulating LDL‐C into cells, thereby reducing plasma concentrations of LDL‐C.^15^ Therefore, loss of activity in the functionally important receptor protein, such as that seen in some cases of HoFH or compound heterozygous FH, will result in ineffectiveness when using PCSK9i.^16^

Based on the two mutation sites identified through the patient's genetic testing, we conducted a comprehensive search for case reports documenting LDLR mutations by using the online database (http://www.ucl.ac.uk/fh). Our analysis revealed that since 1997, a total of 2950 cases have been reported with the specific mutation NM_000527; c.1879G>A; p.Ala627Thr. However, no previous reports were found regarding the other mutation observed in this patient, NM_000527; c.313+1_313+2insT. Notably, c.1879G>A represents one of the most prevalent mutations among Chinese patients diagnosed with FH.^17^ This mutation is associated with impaired binding between LDL and its receptor, which belongs to defective mutation.^17^ The c.313+1_313+2insT mutation has not been previously reported, and no genetic studies have been conducted on this specific mutation. Similar mutations such as c.313+2dupT, are believed to result in a severe clinical phenotype. Previous study has shown that FH patients with mutations exceeding 10 kb exhibit poor responses to drug intervention.^18^ The wide range of frameshift mutations caused by base insertion in the c.313+1_313+2insT mutation may belong to LDLR negative mutations, which can significantly impact the expression and activity of LDLR and render PCSK9i ineffective. In 2017, the clinical guidance for PCSK9i form ESC/EAS emphasizes that treatment with a PCSK9i is not recommended in patients with negative/negative LDLR mutations which have LDL receptor activity below 2%.^19^ It should be noted that this patient has an LDLR defective mutation rather than being exclusively LDLR negative/negative. However, despite this distinction, the patient exhibits no response to PCSK9 inhibitors. While there may exist other undiscovered mechanisms, there is no doubt that this case further demonstrates the necessity for meticulous evaluation prior to initiating treatment with PCSK9i in FH patients. The importance of genetic testing for patients with FH is also highlighted.

Lifestyle intervention and drug therapy for FH patients should ideally commence within the first year of life or at the time of initial diagnosis. In this particular case, the delayed initiation of therapy may have compromised the efficacy of lipid‐lowering treatment, and undoubtedly contributing to the patient's poor prognosis. Therefore, we hope that FH can attract multidisciplinary attention, particularly from dermatology, pediatrics, and cosmetic surgery departments, to expedite early diagnosis and prompt initiation of therapy since patients often initially present to these specialties. In China, there are an estimated 3.8 million patients with HeFH and 1400 patients with HoFH. However, diagnosis and therapeutic rates among these patients remain alarmingly low (less than 5% for HoFH and less than 1% for HeFH). This highlights an urgent need to enhance awareness regarding FH among Chinese healthcare professionals as well as the general public.^20^

## 4 CONCLUSION

Standard lipid‐lowering therapy, including PCSK9i, significantly improves the prognosis of patients with FH. However, the efficacy of PCSK9i depends on LDLR activity. The presence of a wide range of LDLR mutations suggests a potential for inefficacy of PCSK9i treatment. Early identification and genetic testing are essential for FH patients, which can help clinicians better assess their condition and select appropriate management strategies. As achieving target LDL‐C levels remains a challenge for most patients, plasma exchange and lipoprotein apheresis are recommended interventions when feasible.

## AUTHOR CONTRIBUTIONS

**Ziyue Zhang:** Writing – original draft; writing – review and editing. **Rongpei Yang:** Writing – original draft. **Jun Zhu:** Writing – review and editing. **XiaoLi Yang:** Writing – review and editing. **Hao Luo:** Review. **Hongyong Wang:** Writing – review and editing. **Xiaoli Luo:** Supervision; writing – review and editing.

## FUNDING INFORMATION

This work was supported by Chongqing scientific research institutions performance incentive and guidance project (cstc2022ycjh‐bgzxm0106 and cstc2018jscx‐mszdX0024).

## CONFLICT OF INTEREST STATEMENT

The authors have no conflict of interest to declare.

## CONSENT

Written informed consent was obtained from the patient to publish this report in accordance with the journal's patient consent policy.

## Data Availability

All the required information is available in the manuscript itself.
